# Supplementary material for: LTA4H extensively associates with mRNAs and lncRNAs indicative of its novel regulatory targets
Source: PeerJ. 2023 Mar 10;11:e14875. doi: 10.7717/peerj.14875 (PMC10010175; doi:10.7717/peerj.14875)
Supplement: File S2 [file peerj-11-14875-s005.zip › Raw data files/Quality_control/FastqcView/LTA4H_IP_2_fastqc/fastqc_report.html]

E743-IP-2-mRNA\_all.fq.clean\_fq\_reverse FastQC Report


FastQC Report

Mon 16 Oct 2017  
E743-IP-2-mRNA\_all.fq.clean\_fq\_reverse

## Summary

- Basic Statistics
- Per base sequence quality
- Per sequence quality scores
- Per base sequence content
- Per base GC content
- Per sequence GC content
- Per base N content
- Sequence Length Distribution
- Sequence Duplication Levels
- Overrepresented sequences
- Kmer Content

## Basic Statistics

| Measure | Value |
| --- | --- |
| Filename | E743-IP-2-mRNA\_all.fq.clean\_fq\_reverse |
| File type | Conventional base calls |
| Encoding | Sanger / Illumina 1.9 |
| Total Sequences | 27878730 |
| Sequence length | 16-147 |
| %GC | 50 |

## Per base sequence quality

## Per sequence quality scores

## Per base sequence content

## Per base GC content

## Per sequence GC content

## Per base N content

## Sequence Length Distribution

## Sequence Duplication Levels

## Overrepresented sequences

| Sequence | Count | Percentage | Possible Source |
| --- | --- | --- | --- |
| TGGTAGTAGCAAATATTCAAACGAGAACTTTGAAGGCCGAAGTGGAGAAG | 329604 | 1.182277671902558 | No Hit |
| TGGTGGTAGTAGCAAATATTCAAACGAGAACTTTGAAGGCCGAAGTGGAG | 202344 | 0.7258006372600186 | No Hit |
| CCTATTCTCAAACTTTAAATGGGTAAGAAGCCCGGCTCGCTGGCGTGGAG | 121120 | 0.43445307587540755 | No Hit |
| AATATTCAAACGAGAACTTTGAAGGCCGAAGTGGAGAAGGGTTCCATGTG | 119020 | 0.42692045154137226 | No Hit |
| AGCCTAGGGCGCGGGCCCGGGTGGAGCCGCCGCAGGTGCAGATCTTGGTG | 114447 | 0.4105172653130182 | No Hit |
| ATCAGACGTGGCGACCCGCTGAATTTAAGCATATTAGTCAGCGGAGGAGA | 86798 | 0.311341298545522 | No Hit |
| CAGCAGGACGGTGGCCATGGAAGTCGGAATCCGCTAAGGAGTGTGTAACA | 86661 | 0.3108498844818254 | No Hit |
| GTCCGCCGAGGGCGCACCACCGGCCCGTCTCGCCCGCCGCGCCGGGGAGG | 86123 | 0.3089200978667249 | No Hit |
| ACTTTAAATGGGTAAGAAGCCCGGCTCGCTGGCGTGGAGCCGGGCGTGGA | 84523 | 0.30318095551698376 | No Hit |
| GGCCCTTGAAAATCCGGGGGAGAGGGTGTAAATCTCGCGCCGGGCCGTAC | 81931 | 0.293883544910403 | No Hit |
| CCTTGAAAATCCGGGGGAGAGGGTGTAAATCTCGCGCCGGGCCGTACCCA | 80706 | 0.28948951404888246 | No Hit |
| TGAAGCCTAGGGCGCGGGCCCGGGTGGAGCCGCCGCAGGTGCAGATCTTG | 78764 | 0.2825236300218841 | No Hit |
| CAGATCAGACGTGGCGACCCGCTGAATTTAAGCATATTAGTCAGCGGAGG | 75948 | 0.2724227394863396 | No Hit |
| ATTTTGTTGGTTTTCGGAACTGAGGCCATGATTAAGAGGGACGGCCGGGG | 71565 | 0.25670107641201734 | No Hit |
| AGACAGCAGGACGGTGGCCATGGAAGTCGGAATCCGCTAAGGAGTGTGTA | 71045 | 0.25483585514835144 | No Hit |
| GGCGTGGAGCCGGGCGTGGAATGCGAGTGCCTAGTGGGCCACTTTTGGTA | 56582 | 0.20295759527065976 | No Hit |
| TTGGTTTTCGGAACTGAGGCCATGATTAAGAGGGACGGCCGGGGGCATTC | 55782 | 0.20008802409578916 | No Hit |
| TGGCGTGGAGCCGGGCGTGGAATGCGAGTGCCTAGTGGGCCACTTTTGGT | 55459 | 0.19892943473393518 | No Hit |
| TGGAATGCGAGTGCCTAGTGGGCCACTTTTGGTAAGCAGAACTGGCGCTG | 52612 | 0.18871734831536444 | No Hit |
| CTTTAAATGGGTAAGAAGCCCGGCTCGCTGGCGTGGAGCCGGGCGTGGAA | 50841 | 0.18236483512699467 | No Hit |
| TAGTGACGCGCATGAATGGATGAACGAGATTCCCACTGTCCCTACCTACT | 49739 | 0.17841200083361042 | No Hit |
| GCCATGATTAAGAGGGACGGCCGGGGGCATTCGTATTGCGCCGCTAGAGG | 43351 | 0.15549847500226877 | No Hit |
| TGGCCCTTGAAAATCCGGGGGAGAGGGTGTAAATCTCGCGCCGGGCCGTA | 41011 | 0.14710497931577227 | No Hit |
| CTACGGGGTCTGACGCTCAGTGGAACGAAAACTCACGTTAAGGGATTTTG | 39982 | 0.14341399339209498 | No Hit |
| GCATTGAATGAATTTAAATCATCTTCTTTGGCAGTAATCCATCTTTGACT | 39210 | 0.14064485720834485 | No Hit |
| TGGTTTTCGGAACTGAGGCCATGATTAAGAGGGACGGCCGGGGGCATTCG | 38256 | 0.13722289358231168 | No Hit |
| TGGTAGTAGCAAATATTCAAACGAGAACTTTGAAGGCCGA | 35871 | 0.12866798451722872 | No Hit |
| GTGGCATTGAATGAATTTAAATCATCTTCTTTGGCAGTAATCCATCTTTG | 35852 | 0.12859983220182555 | No Hit |
| ACCGTCGTAGTTCCGACCATAAACGATGCCGACCGGCGATGCGGCGGCGT | 34777 | 0.1247438459355932 | No Hit |
| TATAGGGGCGAAAGACTAATCGAACCATCTAGTAGCTGGTTCCCTCCGAA | 34466 | 0.12362830014136227 | No Hit |
| GGAGCCCCGCGGACGCTACGCCGCGACGAGTAGGAGGGCCGCTGCGGTGA | 34281 | 0.12296471180717342 | No Hit |
| AGCTGGTTCCCTCCGAAGTTTCCCTCAGGATAGCTGGCGCTCTCGCAGAC | 34046 | 0.12212177527455517 | No Hit |
| CCGCGACGAGTAGGAGGGCCGCTGCGGTGAGCCTTGAAGCCTAGGGCGCG | 33889 | 0.12155862193148684 | No Hit |
| GTGGAGCCGGGCGTGGAATGCGAGTGCCTAGTGGGCCACTTTTGGTAAGC | 33702 | 0.12088785966936084 | No Hit |
| AATTAGTGACGCGCATGAATGGATGAACGAGATTCCCACTGTCCCTACCT | 32789 | 0.11761296156603979 | No Hit |
| AGGTGAACAGCCTCTGGCATGTTGGAACAATGTAGGTAAGGGA | 31718 | 0.11377132315568177 | No Hit |
| AGGTGAACAGCCTCTGGCATGTTGGAACAATGTAGGTAAGGGAAGTCGGC | 31005 | 0.11121381784607837 | No Hit |
| TTTCTACGGGGTCTGACGCTCAGTGGAACGAAAACTCACGTTAAGGGATT | 30250 | 0.10850566004979424 | No Hit |
| GATCTTGGTGGTAGTAGCAAATATTCAAACGAGAACTTTGAAGGCCGAAG | 29163 | 0.10460663021593883 | No Hit |
| CGGCGGGAGTAACTATGACTCTCTTAAGGTAGCCAAATGCCTCGTCATCT | 29130 | 0.1044882604049754 | No Hit |
| TTGGCAGTAATCCATCTTTGACTTAAGGCAATACAAGCATTTGTCAGAGT | 28812 | 0.10334760586296435 | No Hit |
| ACGATGCCGACCGGCGATGCGGCGGCGTTATTCCCATGACCCGCCGGGCA | 28572 | 0.10248673451050316 | No Hit |
| CCAAGGTGAACAGCCTCTGGCATGTTGGAACAATGTAGGTAAGGGAAGTC | 28411 | 0.10190923331156046 | No Hit |
| GATACCGTCGTAGTTCCGACCATAAACGATGCCGACCGGCGATGCGGCGG | 28052 | 0.10062151324683728 | No Hit |

## Kmer Content

| Sequence | Count | Obs/Exp Overall | Obs/Exp Max | Max Obs/Exp Position |
| --- | --- | --- | --- | --- |
| ACTTT | 5685615 | 3.1037042 | 8.035385 | 25-29 |
| CTTTG | 5697360 | 2.9940479 | 8.3173685 | 25-29 |
| CGCCG | 7339550 | 2.8157032 | 6.176498 | 4 |
| TGAAC | 8269285 | 2.8079114 | 10.260107 | 70-74 |
| TTTTT | 3951280 | 2.7626958 | 8.846275 | 130-134 |
| TTCCC | 4032445 | 2.583397 | 5.531277 | 7 |
| TTGAA | 7338085 | 2.5654037 | 7.725887 | 30-34 |
| TTTTC | 3557665 | 2.4160326 | 8.478396 | 130-134 |
| AACTT | 5473460 | 2.4017622 | 9.1012335 | 25-29 |
| TTGGT | 5684955 | 2.3802195 | 5.8247848 | 45-49 |
| GTTTT | 4281900 | 2.3167498 | 6.638404 | 105-109 |
| TTTTG | 4199790 | 2.2723236 | 5.508881 | 2 |
| GTTCC | 4436485 | 2.26447 | 6.551653 | 100-104 |
| CTGGC | 5711455 | 2.2559097 | 6.046096 | 85-89 |
| GAACT | 6583810 | 2.2355928 | 7.128704 | 25-29 |
| TATTC | 3910110 | 2.134479 | 14.9417305 | 3 |
| TCCCT | 3303725 | 2.1165404 | 5.9646616 | 8 |
| CCCTC | 3377720 | 2.101787 | 10.345383 | 140-142 |
| GAACA | 7505840 | 2.0487072 | 6.0309925 | 60-64 |
| CCATG | 4934390 | 2.0245392 | 8.589105 | 55-59 |
| TGTTG | 4814300 | 2.0156872 | 6.7344594 | 5 |
| GGCGC | 6457310 | 1.9736673 | 5.527141 | 90-94 |
| TGGTT | 4711015 | 1.9724429 | 5.0626435 | 130-134 |
| TGGTG | 6075605 | 1.9684612 | 12.333579 | 1 |
| CCTCC | 3151005 | 1.9607136 | 6.6785803 | 120-124 |
| ATATT | 4306220 | 1.9454578 | 6.6642246 | 2 |
| GGTGG | 7748195 | 1.9426084 | 9.798475 | 2 |
| TTTGA | 4454070 | 1.937157 | 5.695479 | 25-29 |
| GGTTC | 4758275 | 1.9350046 | 7.082924 | 50-54 |
| GCCGC | 5031930 | 1.930421 | 5.3096848 | 25-29 |
| GGCCG | 6252220 | 1.910982 | 6.577465 | 35-39 |
| ATTTT | 3360615 | 1.8887705 | 5.3310833 | 1 |
| GCCTC | 3809010 | 1.8883486 | 5.3328114 | 120-124 |
| AGCAG | 7104050 | 1.8666755 | 7.4595475 | 2 |
| CCGCC | 3875780 | 1.8662578 | 6.6504645 | 3 |
| CCTCT | 2870130 | 1.8387566 | 6.127638 | 80-84 |
| TTTGT | 3374790 | 1.825952 | 5.3535542 | 3 |
| AAATA | 6226780 | 1.8176885 | 6.0088263 | 10-14 |
| TTCAA | 4137560 | 1.8155675 | 8.545052 | 15-19 |
| TCCTG | 3489630 | 1.7811764 | 5.264717 | 85-89 |
| TCTCC | 2779925 | 1.7809666 | 7.1572022 | 8 |
| TGAAG | 6537235 | 1.7685379 | 6.2333045 | 30-34 |
| GGCCC | 4593175 | 1.7620995 | 5.4321156 | 1 |
| ATTCA | 3982835 | 1.7476737 | 8.421062 | 15-19 |
| TCAAA | 4916075 | 1.7340108 | 5.2716255 | 6 |
| CATGT | 4088195 | 1.7269583 | 6.9914384 | 55-59 |
| TCCAT | 3223185 | 1.7089548 | 5.4270325 | 50-54 |
| CAGCA | 5181365 | 1.7088428 | 8.916463 | 1 |
| AGCCT | 4120270 | 1.6905127 | 6.920407 | 1 |
| CTCCA | 3238130 | 1.6675625 | 6.1374664 | 9 |
| TTCCA | 3106370 | 1.6470188 | 5.499193 | 50-54 |
| CAGCC | 4130595 | 1.6460683 | 5.3433013 | 8 |
| CCAAG | 4911955 | 1.6199896 | 5.8302655 | 1 |
| CAAAT | 4557675 | 1.607595 | 6.981224 | 10-14 |
| TTGTT | 2946865 | 1.5944204 | 5.861948 | 4 |
| GCCCT | 3185070 | 1.579025 | 8.359494 | 140-142 |
| GCCGA | 4950855 | 1.5718834 | 5.243743 | 35-39 |
| CAAAC | 4567915 | 1.5649259 | 5.8018603 | 7 |
| TGTTT | 2865375 | 1.5503298 | 5.144092 | 120-124 |
| GCAGG | 6125770 | 1.5495504 | 6.896995 | 3 |
| GGGTT | 4760195 | 1.5422759 | 5.335369 | 50-54 |
| CGGCC | 4011715 | 1.5390315 | 5.002261 | 135-139 |
| ATTCT | 2810195 | 1.5340493 | 8.226926 | 4 |
| TGGTA | 4529485 | 1.5244175 | 17.294352 | 1 |
| AATAT | 4153000 | 1.5081784 | 5.1890736 | 1 |
| TCAGT | 3537175 | 1.4941933 | 8.16557 | 80-84 |
| GAAAG | 6844430 | 1.4884108 | 5.958818 | 140-142 |
| GCAAA | 5295125 | 1.445296 | 13.9557085 | 9 |
| GTCAG | 4403145 | 1.4393308 | 6.3574047 | 80-84 |
| TTCTC | 2178890 | 1.4371943 | 9.255069 | 5 |
| TAGTA | 4102740 | 1.4343227 | 17.771713 | 4 |
| CTTTA | 2617830 | 1.4290398 | 5.5083284 | 2 |
| GCAGT | 4309910 | 1.4088535 | 6.0364356 | 65-69 |
| ATGGG | 5402770 | 1.4070807 | 5.1204095 | 75-79 |
| AATCC | 3274480 | 1.395574 | 5.0392594 | 8 |
| TTGCC | 2725520 | 1.3911595 | 6.5335717 | 125-129 |
| ACTCT | 2595785 | 1.3763031 | 6.0247636 | 120-124 |
| AACGA | 5039875 | 1.375626 | 6.306294 | 20-24 |
| ACCCC | 2707530 | 1.3542647 | 5.8342433 | 115-119 |
| GTAGC | 4137555 | 1.3525128 | 16.429533 | 6 |
| CATGG | 4114125 | 1.3448538 | 5.783602 | 75-79 |
| CCTTG | 2621720 | 1.3381778 | 6.121865 | 4 |
| ATGCC | 3260605 | 1.3377992 | 7.496372 | 140-142 |
| TTTCA | 2445195 | 1.3348006 | 5.499264 | 130-134 |
| CAGTC | 3247965 | 1.3326131 | 5.0457573 | 80-84 |
| TCAGA | 3920045 | 1.3310871 | 5.577161 | 5 |
| TTAAT | 2923885 | 1.3209486 | 5.208549 | 135-139 |
| TCATT | 2384330 | 1.3015752 | 5.9709587 | 125-129 |
| CGCCC | 2701700 | 1.3009173 | 5.090059 | 120-124 |
| GTGGT | 3940625 | 1.2767397 | 12.089501 | 3 |
| ACGAG | 4819485 | 1.2663783 | 5.9537807 | 20-24 |
| GTCTC | 2451610 | 1.2513503 | 5.5302815 | 7 |
| CCCTT | 1946110 | 1.2467806 | 7.748418 | 3 |
| CGAAA | 4563405 | 1.2455742 | 6.9441347 | 140-142 |
| AGTAG | 4535690 | 1.2270539 | 14.069886 | 5 |
| GTAGT | 3632195 | 1.222431 | 17.067541 | 3 |
| CTCAT | 2282980 | 1.2104517 | 6.931454 | 135-139 |
| AGCAA | 4412175 | 1.2042962 | 13.890688 | 8 |
| TCCGC | 2420670 | 1.2000673 | 5.740862 | 2 |
| CTATT | 2182975 | 1.1916581 | 8.169131 | 2 |
| CGCTC | 2378125 | 1.1789753 | 6.0248685 | 135-139 |
| CAGAC | 3540700 | 1.1677424 | 5.647619 | 3 |
| TAGCA | 3409660 | 1.1577812 | 17.009407 | 7 |
| TTCAT | 2118385 | 1.1563993 | 5.1781535 | 125-129 |
| CCTAG | 2812930 | 1.1541219 | 6.1034155 | 3 |
| GCTCA | 2809415 | 1.1526798 | 5.5675464 | 135-139 |
| CAGAT | 3368475 | 1.1437966 | 5.158718 | 1 |
| CCCCA | 2286235 | 1.1435394 | 5.138334 | 115-119 |
| GCCTA | 2771850 | 1.1372672 | 6.451765 | 2 |
| CGGTC | 2850300 | 1.1258111 | 6.610227 | 85-89 |
| TCTCA | 2117930 | 1.122941 | 7.802507 | 6 |
| CGATG | 3361915 | 1.0989662 | 5.8277855 | 125-129 |
| AGGGC | 4298635 | 1.0873655 | 5.9215317 | 9 |
| CGTGG | 3285760 | 1.0339884 | 5.4443226 | 7 |
| CCGAC | 2532220 | 1.0091056 | 5.61146 | 130-134 |
| TGCCG | 2515285 | 0.9934869 | 6.166097 | 140-142 |
| GGTAG | 3743990 | 0.9750733 | 13.220025 | 2 |
| CTCCG | 1959910 | 0.9716417 | 5.1927595 | 120-124 |
| CCGAT | 2367710 | 0.9714517 | 8.299319 | 135-139 |
| TATAG | 2768760 | 0.96796197 | 6.0414186 | 140-142 |
| TCGGT | 2376120 | 0.96627516 | 7.39422 | 85-89 |
| TGCCC | 1911140 | 0.9474636 | 8.5085745 | 140-142 |
| TCCGT | 1754805 | 0.89568734 | 7.3568854 | 135-139 |
| CCTAT | 1678870 | 0.8901484 | 8.030826 | 1 |
| CTCAA | 2061320 | 0.8785288 | 6.2773266 | 7 |
| GATGC | 2644970 | 0.86460626 | 5.375063 | 140-142 |
| TCAAG | 2332855 | 0.79214233 | 5.161051 | 140-142 |
| CCTCG | 1586130 | 0.7863371 | 5.3677993 | 130-134 |
| CTAGG | 2371910 | 0.7753465 | 6.216108 | 4 |
| TCGAA | 1694705 | 0.57545257 | 5.0879 | 140-142 |
| ATCGA | 1355025 | 0.46011114 | 6.3561215 | 140-142 |
| GATCG | 1255300 | 0.4103412 | 6.2814384 | 140-142 |

Produced by FastQC (version 0.9.5)
